# Evaluating the Role of Gracilis Release in Correcting Spastic In-Toeing Gait in Spastic Cerebral Palsy: A Case Report

**DOI:** 10.7759/cureus.49802

**Published:** 2023-12-01

**Authors:** Saksham Goyal, Ratnakar Ambade, Suhas Landge, Adarsh Jayasoorya, Rohan Chandanwale

**Affiliations:** 1 Department of Orthopaedics, Jawaharlal Nehru Medical College, Datta Meghe Institute of Higher Education and Research, Wardha, IND

**Keywords:** rehabilitation, spasticity, gracilis release, adductor tenotomy, cerebral palsy

## Abstract

Cerebral palsy (CP) encompasses a range of conditions that impact an individual's mobility, balance, and posture, making it the most prevalent motor impairment in children. In spastic cerebral palsy, muscle stiffness hinders walking and, if left untreated, may lead to complications such as hip dislocations or dysplasia. Adductor spasticity is a common challenge in these children, significantly impeding mobility and daily activities. The risk of hip dislocation escalates as gross motor function declines, particularly in children with severe impairments. This case report highlights the successful application of bilateral adductor tenotomy with gracilis release in a 9-year-old child diagnosed with spastic cerebral palsy, exhibiting a scissoring and in-toeing gait. Additionally, this report prompts consideration of the potential benefits of gracilis release in addressing the in-toeing gait observed in children affected by spastic cerebral palsy.

## Introduction

The most prevalent cause of motor impairment among children in Europe, affecting approximately 700,000 individuals, is cerebral palsy (CP). The prevalence of CP in Europe has remained constant over the past 30 years, ranging between 1.5 and 3 instances per 1000 live births. CP manifests as complex motor abnormalities, involving not only secondary issues like muscular contractures and skeletal deformities but also fundamental deficits such as muscle stiffness, weakness, and the loss of selective motor control. These impairments lead to restricted activities, including challenges in posture and movement during activities like walking [1]. In children with CP, the risk of hip displacement increases with declining gross motor ability, reaching up to 90% in the most severely affected cases [2]. Spastic cerebral palsy, a prevalent form characterized by muscle stiffness and tightness, results in gait abnormalities, muscle contractures, and other impairments. The scissoring gait that adductor spasticity frequently exhibits makes it difficult to move, go through daily activities, and maintain personal hygiene. Hip subluxation and dislocation can occur due to factors such as coxa valga, pelvic obliquity, and acetabular shallowness resulting from a lack of weight-bearing [3].

Various treatments, including physiotherapy exercises and surgeries, have been explored. One surgical intervention is adductor tenotomy, a minimally invasive procedure that releases muscle contractures and improves gait in affected children. When a patient's adductors become overactive, their gait becomes narrower, which can lead crossing of legs and falling. It is recognized that releasing the gracilis, or adductor longus, can widen the gait [4]. So an attempt was made by releasing the gracilis and correcting the in-toeing gait. While conclusive evidence supporting the efficacy of gracilis release is currently lacking, further studies are warranted to establish its inclusion in routine procedures. Adductor tenotomy involves cutting the tendon of the adductor muscle to alleviate spasticity, while gracilis release entails releasing fibers at its insertion point to correct in-toeing gait. These procedures are commonly performed in individuals with cerebral palsy, specifically addressing spasticity in the hip adductor muscles, which can lead to a crossing or scissoring gait during walking [5]. In this report, we present a case involving a 9-year-old female child diagnosed with spastic cerebral palsy, exhibiting a scissoring and in-toeing gait. The child was successfully managed with a combination of adductor tenotomy and gracilis release.

## Case presentation

**Figure 1:**
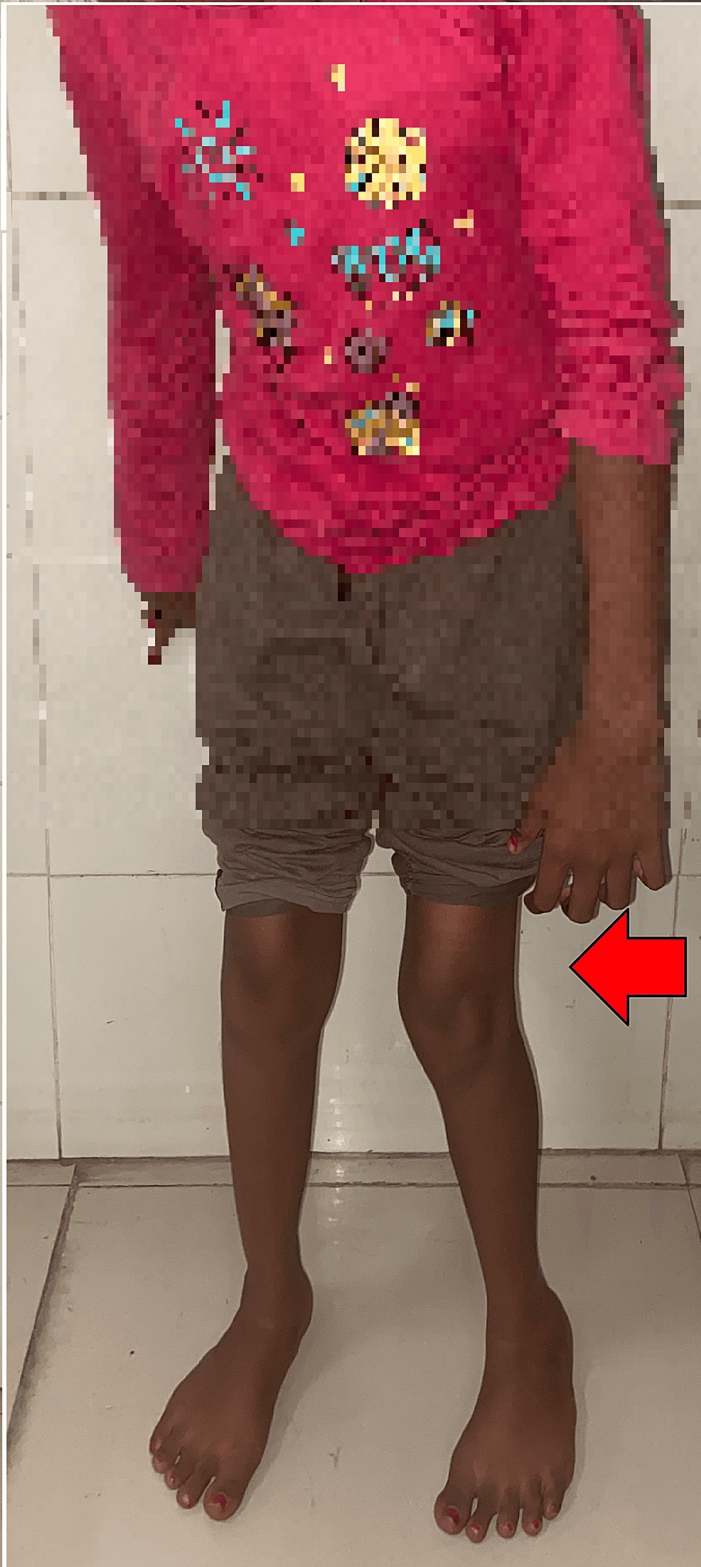
Pre-operative image of the female with spastic cerebral palsy

A 9-year-old female child diagnosed with spastic diplegia sought treatment at our Acharya Vinoba Bhave Rural Hospital (AVBRH) due to challenges in walking and standing, attributed to tightness in the hip adductor muscles. The patient's history revealed that she commenced walking at the age of two years but experienced progressively restricted hip muscle movements over time, resulting in an inability to walk for the past three years. Upon examination, she exhibited a spastic gait with internal rotation and adduction of the hips, indicative of a pelvic obliquity likely caused by internal rotation, as seen in Figure 1. Efforts to mobilize using a walker were hindered by a scissoring gait and in-toeing. Additionally, the patient demonstrated tightness in the adductor and gracilis muscles, as evidenced by a modified Tardieu score of 3. Following a comprehensive evaluation and discussions with the family, the decision was made to proceed with bilateral adductor tenotomy surgery and gracilis release.

**Figure 2:**
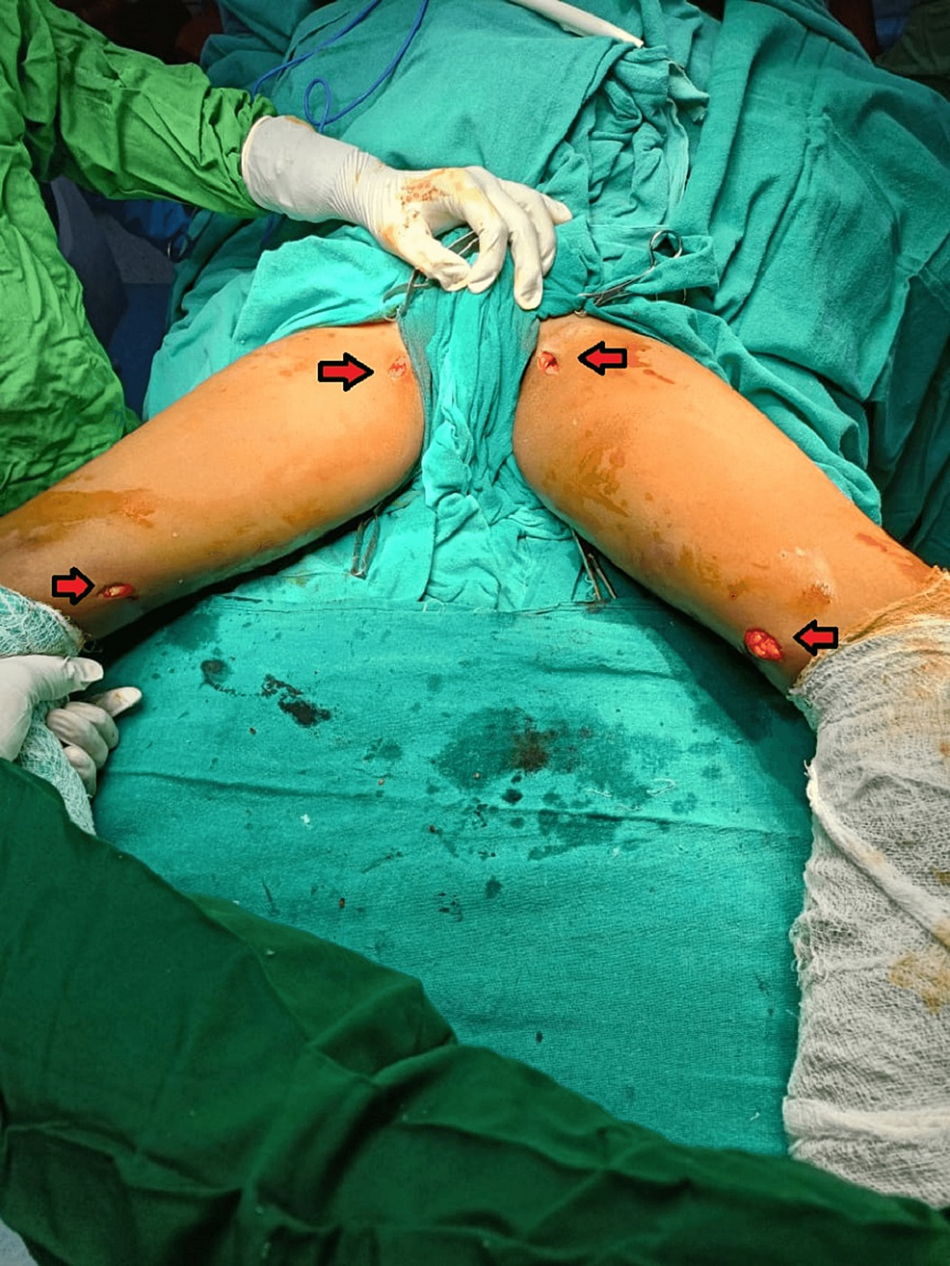
Intra-operative images showing adductor tenotomy and gracilis release

The surgical procedure, conducted under spinal anesthesia with the patient in the supine position, involved a 2 cm incision over the medial aspect of the thigh. The adductor longus tendon was identified and cut using a scalpel, as depicted in Figure 2. Gracilis muscle fibers were released near their insertion point using the same technique.

**Figure 3:**
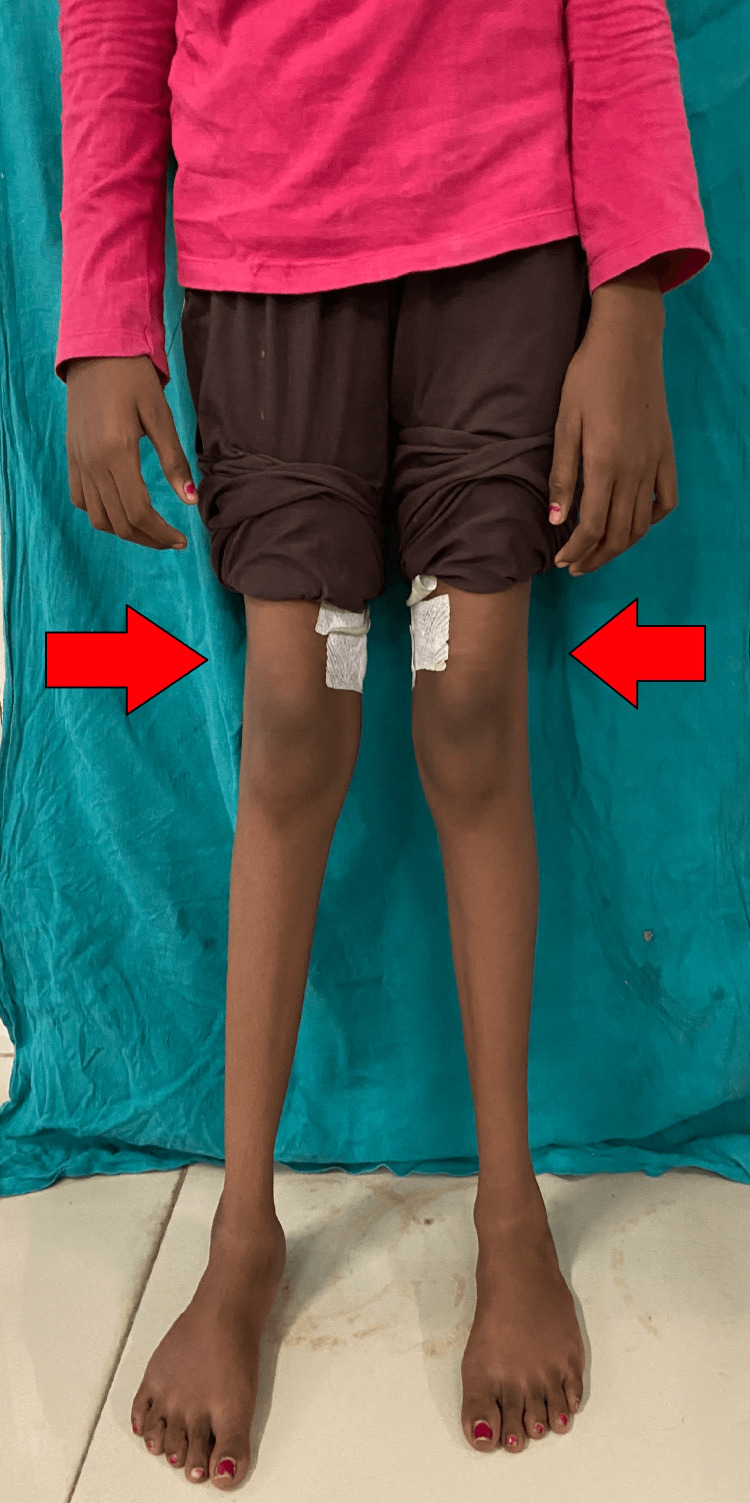
Standing image three days post-surgery The image shows that in-toeing is corrected.

**Figure 4:**
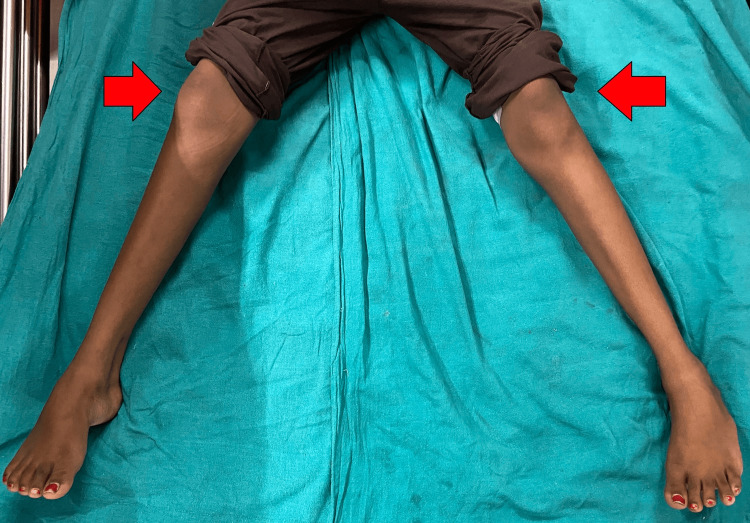
Three days post-surgery image The movement over the hip joint is improved with abduction around 45 degrees.

Postoperatively, the patient underwent a rehabilitation regimen, including hamstring stretching, quadriceps and abductor strengthening exercises, and adductor stretching exercises. Derotation straps were applied to prevent in-toeing gait. Within 2-3 days, the adductor and gracilis muscles showed relaxation, resulting in a significant improvement in hip joint range of motion and correction of pelvic drooping, indicating the absence of hip rotation, as illustrated in Figures 3-4. Gait training was initiated, and within a week, the patient commenced walking with the assistance of a walker.

**Figure 5:**
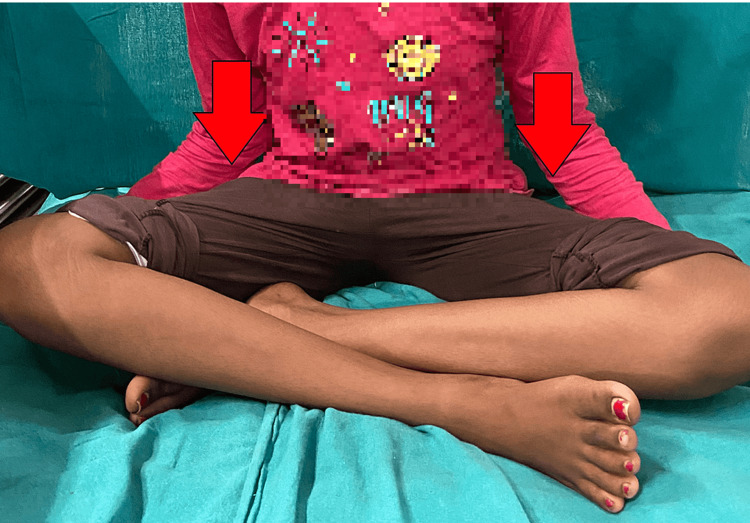
Six weeks post-surgery sitting image The patient is able to sit cross-legged.

**Figure 6:**
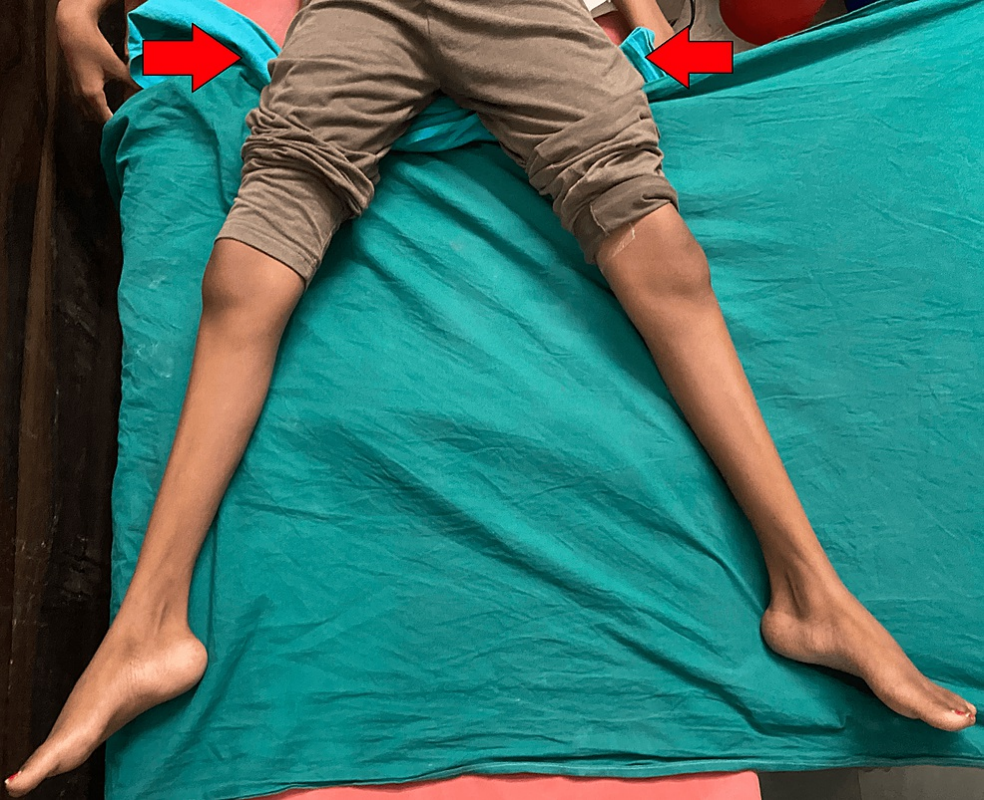
Six weeks post-surgery image The external rotation is significantly improved.

**Figure 7:**
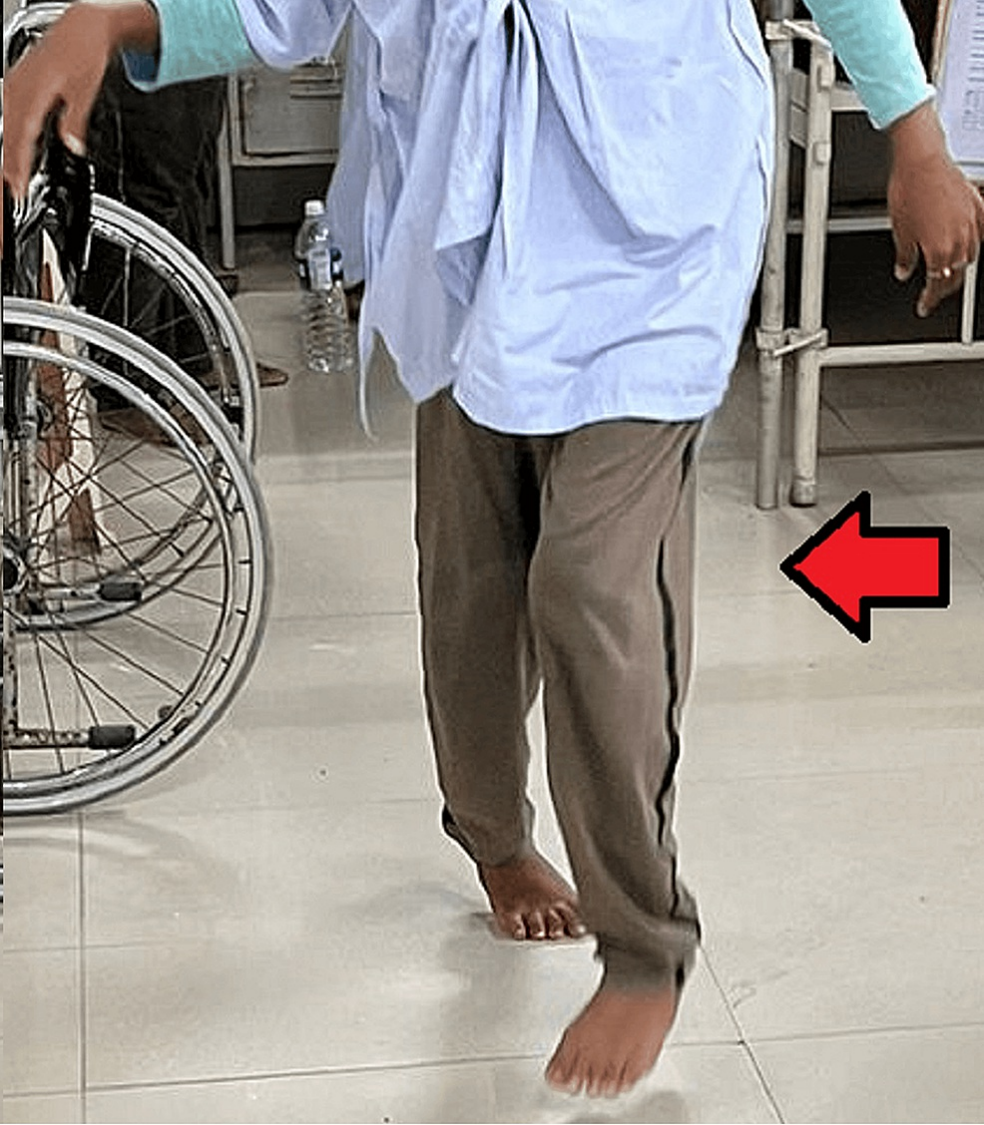
An image two months after the surgery The patient is able to walk without a walker.

After a six-week follow-up, the patient demonstrated the ability to sit cross-legged (Figures 5-6), and within the subsequent two months, she progressed to squatting and walking without a walker, easily engaging in day-to-day activities, as depicted in Figure 7.

## Discussion

Spastic cerebral palsy represents a common cause of muscle spasticity, resulting in challenges for individuals in their daily activities and ambulation. The imbalance among antagonistic muscles often leads to deformities and contractures in affected children [6]. Diverse treatment modalities have been explored, encompassing physiotherapy and surgical interventions [7]. Soft tissue procedures, including adductor tenotomy and obturator neurectomy, are frequently combined in spastic hip surgery. Timely correction of spastic hip muscles is crucial, as delays may lead to complications such as hip dislocation, arthritis, and avascular necrosis, severely impeding future mobility. The effectiveness of interventions must be weighed against the natural progression of the condition, with the understanding that preventative therapies may pose risks of unnecessary surgeries [8]. Prioritizing the restoration of muscular balance is paramount, given that it addresses the core etiology. Unfortunately, for non-ambulatory patients, options for enhancing weight-bearing capabilities are limited [9].

By the age of 10, up to 75% of children with four-limb cerebral palsy may experience hip dislocation, and approximately 50% of these cases result in pain. Surgical intervention is recommended for subluxated hips in children with cerebral palsy to prevent painful hip dislocation [10]. Examination of at-risk hips typically reveals concurrent adduction and flexion contractures, the combination of which precipitates dislocation [11]. The degree of involvement and neurological maturity play pivotal roles in preventing hip instability. Optimal outcomes are observed in diplegic and tetraplegic children who have achieved sufficient neurological development. Conversely, severely impaired, neurologically immature quadriplegic children pose greater challenges, with the best outcome often being a dysplastic or moderately subluxated hip that remains prone to continual flexion and adduction despite attempts at force neutralization or balancing [12].

In the presented case, the implementation of adductor tenotomy and gracilis release resulted in a substantial reduction of spasticity, facilitating the patient's mobilization within a week. Typically conducted as an outpatient procedure, adductor tenotomy can be performed under spinal anesthesia or local anesthesia with sedation. The surgeon makes a small incision in the inner thigh, cutting the adductor muscle tendon to alleviate spasticity. Suturing or surgical glue is then used to close the incision. In this instance, gracilis release was additionally performed due to detected tightness at the insertion site, potentially contributing to excessive internal rotation of the hips. While promising, further studies are warranted for conclusive results. Post-procedure, patients may experience discomfort and swelling, manageable with pain medication and ice packs. Rehabilitation involves physical therapy and stretching exercises to enhance range of motion and bolster hip muscle strength for early mobilization. Demonstrating efficacy in reducing spasticity and enhancing walking ability, adductor tenotomy with gracilis release emerges as a viable treatment for spastic cerebral palsy. Subsequent follow-up visits at one, three, and six months post-surgery exhibited sustained improvements in gait and overall function, underscoring the effectiveness of this intervention for correcting both scissoring and in-toeing gait. Our experiential insights suggest caution in performing unilateral operations on the adductor, gracilis, and obturator, particularly in small children, as this cohort exhibited a higher rate of complications [13].

## Conclusions

Adductor tenotomy emerges as a secure and efficacious surgical intervention for treating hip adductor spasticity in cerebral palsy patients when conservative measures prove inadequate. This procedure proves particularly valuable in addressing challenges associated with heightened adductor tone, hip subluxation or dislocation, and muscle contractures commonly encountered in individuals with spastic cerebral palsy. While this study underscores the proven merits of adductor tenotomy, it places additional emphasis on the significance of gracilis release, a nuanced aspect of the procedure that was implemented to correct the in-toeing gait observed in the patient. Presently, there is a paucity of published studies elucidating the importance of gracilis release, necessitating further investigations to comprehensively assess its impact and outcomes for patients. As this facet of the procedure holds promise in addressing specific gait abnormalities, a broader understanding of its implications could potentially refine and enhance treatment approaches for individuals with spastic cerebral palsy.
